# Combining faecal immunochemical testing with blood test results for colorectal cancer risk stratification: a consecutive cohort of 16,604 patients presenting to primary care

**DOI:** 10.1186/s12916-022-02272-w

**Published:** 2022-03-15

**Authors:** Diana R. Withrow, Brian Shine, Jason Oke, Andres Tamm, Tim James, Eva Morris, Jim Davies, Steve Harris, James E. East, Brian D. Nicholson

**Affiliations:** 1 Nuffield Department of Primary Care Health Sciences, University of Oxford, Radcliffe Observatory Quarter, Oxford, OX2 6GG UK; 2 Department of Clinical Biochemistry, John Radcliffe Hospital, Oxford University Hospitals NHS Foundation, Oxford, UK; 3 Nuffield Department of Population Health, Big Data Institute, University of Oxford, Oxford, UK; 4 Department of Computer Science, Big Data Institute, University of Oxford, Oxford, UK; 5 Oxford BRC Informatics Theme, Big Data Institute, University of Oxford, Oxford, UK; 6 Translational Gastroenterology Unit, and Oxford NIHR Biomedical Research Centre, John Radcliffe Hospital, University of Oxford, Oxford, UK

**Keywords:** Colorectal neoplasms, Predictive value of tests, Primary health care, Triage, Faecal immunochemical tests, Full blood count

## Abstract

**Background:**

Faecal immunochemical tests (FITs) are used to triage primary care patients with symptoms that could be caused by colorectal cancer for referral to colonoscopy. The aim of this study was to determine whether combining FIT with routine blood test results could improve the performance of FIT in the primary care setting.

**Methods:**

Results of all consecutive FITs requested by primary care providers between March 2017 and December 2020 were retrieved from the Oxford University Hospitals NHS Foundation Trust. Demographic factors (age, sex), reason for referral, and results of blood tests within 90 days were also retrieved. Patients were followed up for incident colorectal cancer in linked hospital records. The sensitivity, specificity, positive and negative predictive values of FIT alone, FIT paired with blood test results, and several multivariable FIT models, were compared.

**Results:**

One hundred thirty-nine colorectal cancers were diagnosed (0.8%). Sensitivity and specificity of FIT alone at a threshold of 10 μg Hb/g were 92.1 and 91.5% respectively. Compared to FIT alone, blood test results did not improve the performance of FIT. Pairing blood test results with FIT increased specificity but decreased sensitivity. Multivariable models including blood tests performed similarly to FIT alone.

**Conclusions:**

FIT is a highly sensitive tool for identifying higher risk individuals presenting to primary care with lower risk symptoms. Combining blood test results with FIT does not appear to lead to better discrimination for colorectal cancer than using FIT alone.

**Supplementary Information:**

The online version contains supplementary material available at 10.1186/s12916-022-02272-w.

## Background

Diagnosing colorectal cancer in patients who present to primary care can be challenging because many of the symptoms of colorectal cancer are shared with other, less serious causes. Colonoscopy is the definitive test to diagnose colorectal cancer, but referring all patients with symptoms of possible colorectal cancer for colonoscopy would cause significant strain on health care resources and present unnecessary risks to patients [1]. After recommending that FIT be used in the national bowel cancer screening programme in 2016, in 2017, the faecal immunochemical test (FIT) was recommended by the National Institute for Health and Care Excellence (NICE) as a triage test for patients presenting to primary care with low risk symptoms of possible colorectal cancer [2]. The evidence underpinning that recommendation was drawn primarily from higher risk populations, and there was limited evidence about how it would perform in primary care [3–5].

There has been a rapid increase in publications about FIT use in symptomatic patients over the last 5 years [3, 6, 7]. FIT has consistently been shown to have high sensitivity and specificity for colorectal cancer at a threshold of 10μg Hb/g faeces or lower, in primary and secondary care [7–9]. Despite a high negative predictive value, nearly one in ten colorectal cancers will be missed using FIT alone to select who should be referred for investigation [10]. Developing strategies to identify symptomatic people with FIT-negative colorectal cancer has become an urgent priority due to the increased use of FIT to defer or decline colorectal investigation during the COVID-19 pandemic [11]. Furthermore, as the number of colorectal cancer presentations is expected to increase, and health care resources continue to be strained by ongoing effects of the pandemic, efforts to reduce unnecessary referrals by increasing specificity would be especially worthwhile [11].

Clinical prediction models are one strategy to achieve these aims. However, the faecal haemoglobin age and sex test (FAST) score did not improve utility over FIT alone [12]. FIT has also been shown to outperform multivariable models including age, sex, and symptoms prompting urgent cancer referral [13]. Combining commonly used blood tests with FIT could further optimise the triage of symptomatic patients in primary care for colorectal cancer investigation [14, 15]. Using the largest existing UK cohort of symptomatic patients tested with FIT in primary care [16], the aim of this study was to assess whether complementing FIT with blood test values could improve the predictive performance of FIT.

## Methods

### Study design

#### Population/setting

Data were retrieved from the Oxford University Hospitals NHS Foundation Trust (OUH). OUH serves all 67 General Practice (GP) surgeries in the county of Oxfordshire, UK, with a population of approximately 660,000. Based at the John Radcliffe Hospital, the Clinical Biochemistry Laboratory performs over 8 million tests a year. This study was registered as a service evaluation on the OUH Datix register (CSS-BIO-3 4730).

#### FITs

All consecutive FIT results (measured in μg Hb/g faeces) between March 2017 and December 21, 2020, were retrieved retrospectively from the OUH Clinical Biochemistry Laboratory Information Management System.

**Fig. 1:**
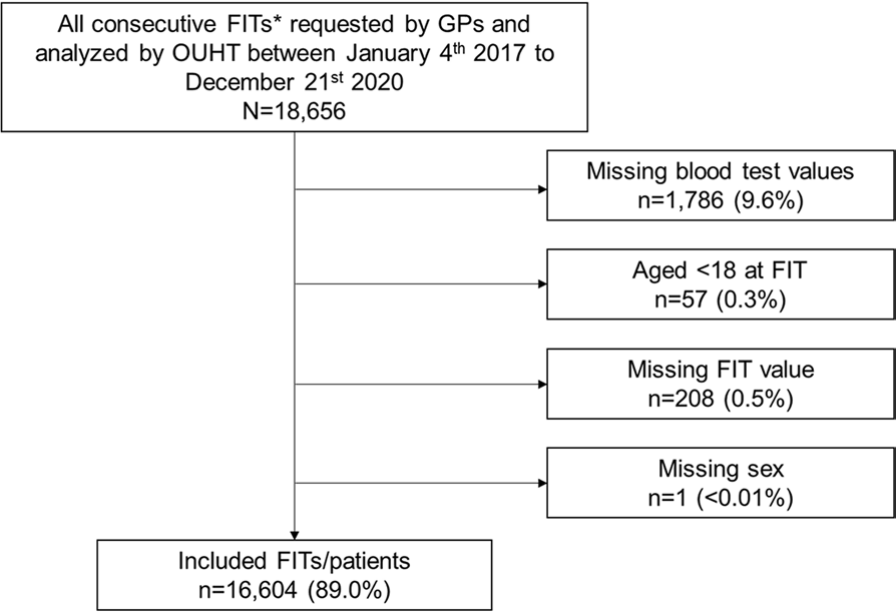
Selection criteria for inclusion. *First FITs per individual

After restricting to FITs requested by primary care clinicians and the first FIT in any given individual, FITs were retained for inclusion in this analysis if the five most common “core” blood tests (haemoglobin, platelets, white cell count, mean cell haemoglobin [MCH], and mean cell volume [MCV]) were available, patients were aged 18 or older, had known sex, and had non-missing FIT results (Fig. 1).

Faecal specimens were collected into standard pots by patients in primary care and referred to the central laboratory where sampling was undertaken using the Extel Hemo-Auto MC device. Prepared samples were analysed for FIT using the HM-JACKarc analyser (Hitachi Chemical Diagnostics Systems Co., Ltd., Tokyo, Japan, and distributed in the UK by Alpha Labs Ltd., Eastleigh, Hants) a method recommended for use by NICE [2]. The method had a calibration range of 7–400 μg Hb/g faeces. For the duration of the study period, 7 μg Hb/g was used to define a positive result in the lab, as this threshold was determined prior to the NICE recommendation to use 10 μg Hb/g faeces. Immunoassay reproducibility assessed across 12 months had a coefficient of variation (CV) of between 4.4 and 8.8%. The overall imprecision of the process including sampling variation was between 7.0 and 13.5 CV% [17]. FIT samples were assayed and recorded prior to and independent of the any subsequent pathology findings.

#### Additional variables

Age, sex, clinical indication, and results of contemporaneous blood tests were retrieved for each patient. To extract the clinical indication, free text fields included in the electronic FIT request were searched for common indications (abdominal pain, bloating, blood in stool, change in bowel habit, constipation, diarrhoea, family history of cancer, fatigue, melaena, rectal pain, and weight loss) using numerous permutations of spelling and phrasing.

Blood test results reported less than 60 days prior to or 30 days post FIT were retrieved. The most routinely used blood tests and those with a hypothesised relationship with colorectal cancer risk were selected for analysis (haemoglobin, platelets, white cell count, MCH, and MCV; serum ferritin; and c-reactive protein [CRP]) [18]. The same analytical methods for the blood tests were used throughout the study period: full blood count, including haemoglobin, platelets, white cell count, MCH, and MCV were analysed using a Sysmex XN analyser (Sysmex UK Ltd, Milton Keynes, UK); ferritin using an Abbott Architect i2000 and CRP using the Abbott Architect c16000 (both Abbott Diagnostics UK, Maidenhead, UK).

#### Outcomes

The composite reference standard incorporated the review of multiple-linked databases (hospital clinical records, pathology results, and endoscopy and radiology reports) for evidence of a new colorectal cancer diagnosis. In the primary analysis, a patient was considered a colorectal cancer case if a diagnosis occurred within 6 months of the FIT. The cut-off date for eligible FITs (December 21, 2020) was selected to allow for at least 6 months follow-up for all patients (until the end of linked clinical records, June 21, 2021). As patients were observed for outcomes through passive linkage rather than active follow-up, patients were not censored or lost to follow-up during that interval. A composite reference standard was used as not all patients tested with FIT in primary care are referred for definitive testing. A reliance on definitive testing alone would lead to verification bias for FIT-positive patients. Database review was independent of FIT value.

#### Patient and public involvement

No patients were directly involved in designing the research question or in conducting the research. A patient advocate provided feedback on interpretation of the results and key messages. Our findings will be disseminated to patients and the public through the NIHR BRC, Nuffield Department of Primary Care Health Sciences, Oxford Cancer, and OxCODE.

### Statistical analysis

Three approaches were investigated to optimise FIT.

1. FIT alone—dichotomous FIT at a cut-off of greater than or equal to 2 or 10 μg Hb/g faeces;

These cut-offs were based on the assays Limit of Detection (2 μg Hb/g faeces) and the cut-off recommended by NICE for primary care triage in 2017 (10 μg Hb/g faeces) and align with existing research [2, 9].

2. FIT-Blood test pairs—dichotomous FIT and dichotomous blood test result;

A test was considered positive if patients fell above the cut-off value for FIT (2 or 10 μg Hb/g faeces) and had an abnormal blood test result. The threshold for abnormal blood tests were pre-specified based on standard clinical practice [19].

3. Multivariable FIT—modelling including FIT, blood tests, age, and sex.

Logistic regression was used to generate predicted probabilities of colorectal cancer. Backward stepwise selection was used to select covariates. Because serum ferritin and CRP were only available for a subset of cases, stepwise selection was conducted on an imputed dataset with 10 replicates using predictive mean matching. In models where CRP or serum ferritin were retained, coefficients for each variable in the imputed and complete case datasets were compared, and if similar, the model results from the complete case dataset were reported. The three modelling approaches are defined below.

- Model A: FIT, age, and blood test results (continuous) and sex (dichotomous).
- Model B: FIT and blood test results (dichotomous), age (categorical), and sex (dichotomous)
- Model C: FIT (spline), age (continuous), sex, and blood tests (dichotomized).

The restricted cubic spline function for FIT was specified to have knots at 2, 10, 50, and 100 μg Hb/g faeces. Four knots were selected to yield a model with at least 20 events per variable, to minimise optimism bias [20]. Ninety-five percent confidence intervals were estimated using the Wilson Score method [21]. The positive predictive value (PPV) and negative predictive value (NPV) were additionally expressed as the number of positive FITs to detect one cancer (number needed to scope) and the cancer miss rate per 10,000 negative tests. To permit a comparison of model performance, the probability cut-off to determine a positive result was selected to match the sensitivity of the FIT alone at a cut-off of 10 μg Hb/g faeces.

#### Sensitivity and subgroup analyses

Each of the approaches 1, 2, and 3 outlined above were replicated with 12 months of follow-up. The FIT alone approach was applied to subgroups defined by FIT date (prior to or during the COVID-19 pandemic), age group (<40, >50, >60, >70, >80), sex, blood test results, and clinical indication (individual symptoms and meeting 2017 NICE DG30 guideline criteria for FIT use [yes vs. no]). The predictive value of abnormal blood tests was estimated in subgroups of FIT-negative patients at thresholds of 2 and 10 μg Hb/g faeces.

All analyses were conducted using Stata version 16.1.

## Results

### Descriptive

**Fig. 2:**
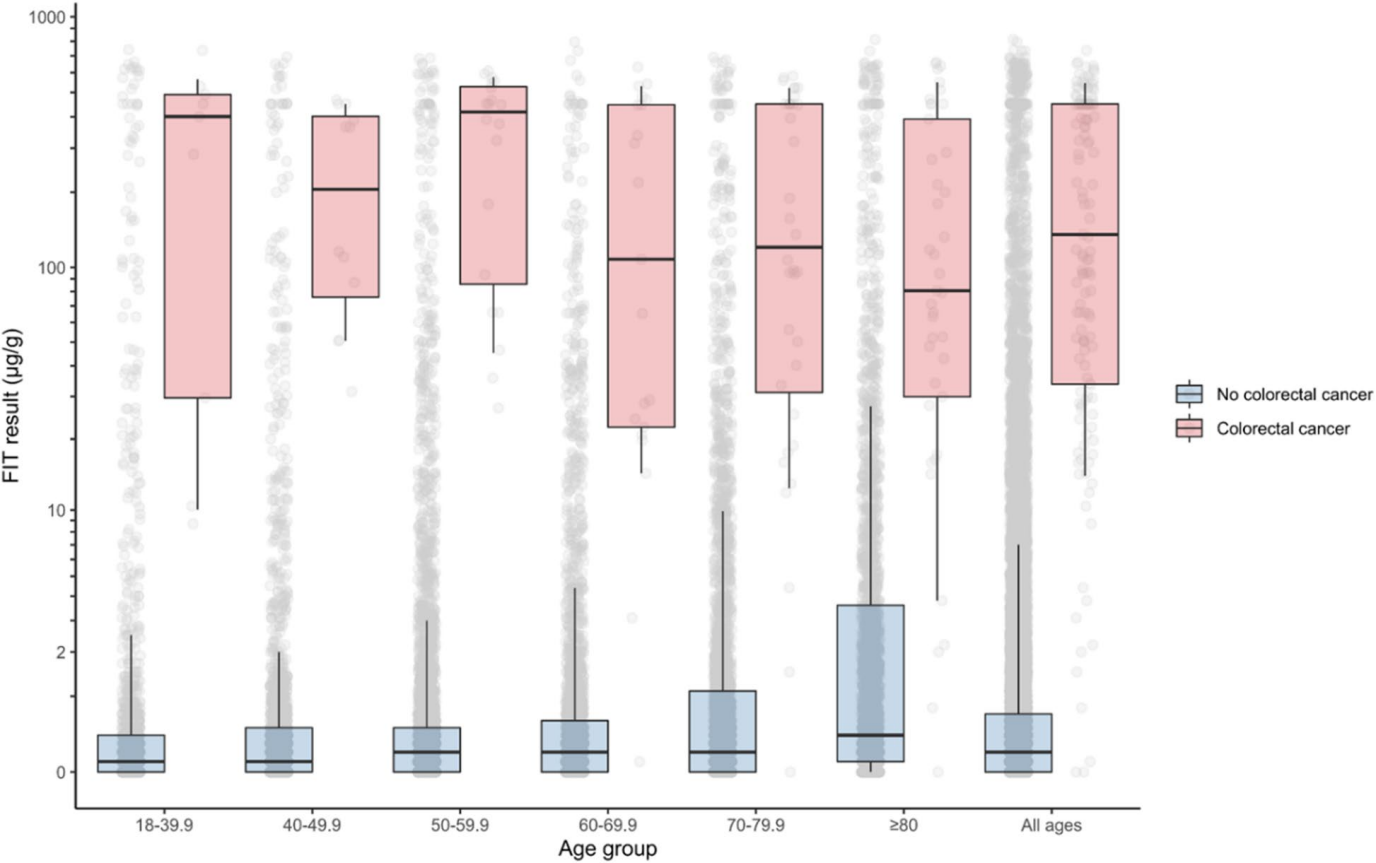
Distribution of FIT score by age and outcome. Boxes indicate median and interquartile range. Whiskers indicate 10th and 90th percentiles. The shape of the distribution corresponds to log10(FIT + 1) whereas tick marks are drawn at actual FIT values

A total of 16,604 of 18,656 available FITs (89%) were included in the study. Included patients were representative of the overall sample (Table 1). Study subjects had a median age of 61 and were 58% female. One-hundred thirty-nine (139) cancers were diagnosed within 6 months of the FIT test (0.8%). Patients who were diagnosed with cancer were older (median age 72) and more likely to be male (60%), to have a FIT ≥10 μg Hb/g faeces, and/or to have abnormal blood tests (Table 1, Fig. 2, Additional file 1: Table S1).

**Table 1:** Characteristics of patients receiving symptomatic FIT tests by study inclusion status and outcome

	All FIT tests		Included		Cancer	
	n	%	n	%	n	%
**Total**	18,656	100	16,604	100	139	100
**Age**						
0-18	95	1	0	0	0	0
18-39.9	1,651	9	1,390	8	9	6
40-49.9	2,553	14	2,278	14	12	9
50-59.9	4,679	25	4,181	25	20	14
60-69.9	3,186	17	2,892	17	21	15
70-79.9	3,711	20	3,330	20	36	26
≥ 80	2,781	15	2,533	15	41	29
Median (IQR)	61	(50, 74)	61	(51, 75)	72	(57, 81)
**Sex**						
Male	7,926	42	7,019	42	83	60
Female	10,728	58	9,585	58	56	40
Unknown	2	0	0	0	0	0
**FIT (µg Hb/g)**						
0-1.9	15,298	82	13,757	83	5	4
2-9.9	1,409	8	1,318	8	6	4
10-99.9	1,072	6	1,023	6	51	37
≥ 100	539	3	506	3	77	55
Missing	338	2	0	0		
Median (IQR)	0	(0, 0.7)	0.2	(0, 0.8)	135.5	(33.4, 450)
Blood test results*						
Low haemoglobin^a^	5,186	31	5,076	31	72	52
High platelets^b^	556	3	546	3	13	9
High white cells^c^	832	5	820	5	9	6
Low mean cell haemoglobin^d^	2,792	17	2,730	16	47	34
Low mean cell volume^e^	1,014	6	980	6	30	22
Any abnormal FBC	6,521	35	6,392	38	81	58
Low serum ferritin^f^	2,015	22	1,962	22	36	40
High serum ferritin^g^	457	5	444	5	3	3
High C-reactive protein^h^	1,748	14	1,720	14	31	31
**Clinical indication - GP reported**						
Abdominal pain	3,299	18	2,941	18	23	17
Blood in stool	1,759	9	1,451	9	22	16
Melaena	298	2	238	1	0	0
Change in bowel habit	7,511	40	6,656	40	45	32
Diarrhoea	2,651	14	2,315	14	13	9
Constipation	722	4	608	4	1	1
Fatigue	199	1	193	1	1	1
Rectal pain	106	1	95	1	0	0
Bloating	594	3	541	3	2	1
Family history of cancer	342	2	263	2	2	1
Weight loss	1,448	8	1,348	8	9	6
**Blood - GP reported**						
Anaemia (any)	4,517	24	4,272	26	48	35
Iron deficiency anaemia	1,926	10	1,793	11	18	13
Thrombocytosis	216	1	204	1	2	1

Any abnormal full blood count (FBC) refers to any abnormal result of heamoglobin, platelets, white cells, mean cell haemoglobin and mean cell volume

*Note*: Serum ferritin and c-reactive protein tests were only conducted for a subset of patients (*n* = 8,922 and 12,201 respectively)

*IQR* Interquartile range

*percent with non-missing values

^a^ <130 g/L in men and <120 g/L in women

^b^ >400 μL/L

^c^ >11,000/mL

^d^ <27.4 pg/cell

^e^ <80 fL

^f^ <20 ng/mL

^g^ ≥350 ng/mL

^h^ >10 mg/L

For 90% of included patients, the free text in the electronic FIT request mapped onto at least one of the pre-specified 11 clinical indication categories. The most common of these was change in bowel habit (40%), then anaemia (26%) and abdominal pain (18%, Table 1). The most common clinical indications in people with cancer were anaemia (35%), change in bowel habit (32%), blood in stool (16%), and abdominal pain (17%).

Low haemoglobin was the most common abnormal blood test result (31% of all patients, 52% of those with a subsequent cancer diagnosis, Table 1) followed by low MCH (16% and 34%, respectively).

#### FIT alone

At a threshold of 2 μg Hb/g faeces, 17.1% of patients would be considered FIT positive. Sensitivity was 96.4% (95% CI 91.9–98.5), specificity 83.5% (95% CI 82.9–84.1), PPV 4.7% (95% CI 4.0–5.5), and NPV 100% (95% CI 99.9–100) (Table 2, Additional file 1: Table S2). One cancer was detected for every twenty-one positive FITs, and the cancer miss rate was 4 cancers per 10,000 negative tests (Table 2).

**Table 2:** Test performance as measured by positive and negative predictive value (PPV, NPV), sensitivity, specificity, positive FITs per cancer detected, and cancer miss rate per 10,000 negative tests. FIT alone and threshold-based approach to FIT-blood test pairs

Test criteria		PPV (95% CI)	NPV (95% CI)	Sensitivity (95% CI)	Specificity (95% CI)	Positive FITs to detect one cancer	Cancer miss rate per 10,000 negative tests
**FIT alone**							
FIT≥2 µg Hb/g		4.7% (4.0, 5.5)	100.0% (99.9, 100)	96.4% (91.9, 98.5)	83.5% (82.9, 84.1)	21	4
FIT≥10 µg Hb/g		8.4% (7.1, 9.9)	99.9% (99.9, 100)	92.1% (86.4, 95.5)	91.5% (91.1, 91.9)	12	7
**FIT-Blood Test Pairs**							
FIT≥2 µg Hb/g AND	Low haemoglobin^a^	5.1% (4.3, 6.2)	99.9% (99.8, 99.9)	89.1% (82.2, 93.5)	83.4% (82.7, 84.0)	17	44
	High platelets^b^	5.8% (4.6, 7.3)	99.6% (99.4, 99.7)	51.1% (42.9, 59.2)	93.0% (92.6, 93.4)	11	77
	High white cells^c^	8.7% (5.2, 14.4)	92.8% (91.5, 93.9)	9.4% (5.5, 15.3)	92.3% (91.0, 93.4)	29	80
	Low mean cell haemoglobin^d^	3.4% (1.7, 6.6)	99.2% (99.1, 99.3)	5.8% (2.9, 10.9)	98.6% (98.4, 98.8)	12	57
	Low mean cell volume^e^	8.0% (6.1, 10.5)	99.4% (99.3, 99.5)	33.8% (26.5, 42.0)	96.7% (96.4, 97.0)		66
	Any abnormal FBC	15.9% (11.4, 21.8)	99.3% (99.2, 99.4)	21.6% (15.6, 29.1)	99.0% (98.9, 99.2)	19	40
	Low serum ferritin^f^	5.4% (4.3, 6.7)	99.6% (99.5, 99.7)	56.8% (48.5, 64.8)	91.6% (91.1, 92.0)	10	63
	High serum ferritin^g^	10.0% (7.3, 13.6)	99.4% (99.2, 99.5)	40.0% (30.5, 50.3)	96.3% (95.9, 96.7)	33	99
	High C-reactive protein^h^	3.0% (1.0, 8.5)	99.0% (98.8, 99.2)	3.3% (1.1, 9.3)	98.9% (98.7, 99.1)	18	59
FIT≥10 µg Hb/g AND	Low haemoglobin^a^	5.6% (4.0, 7.8)	99.4% (99.3, 99.5)	31.0% (22.8, 40.6)	95.7% (95.3, 96.0)	10	44
	High platelets^b^	10.3% (8.2, 12.8)	99.6% (99.4, 99.7)	49.6% (41.5, 57.8)	96.4% (96.1, 96.6)	8	77
	High white cells^c^	12.2% (7.1, 20.2)	99.2% (99.1, 99.4)	8.6% (5.0, 14.5)	99.5% (99.4, 99.6)	17	80
	Low mean cell haemoglobin^d^	5.8% (2.9, 10.9)	99.2% (99.1, 99.3)	5.8% (2.9, 10.9)	99.2% (99.1, 99.3)	7	58
	Low mean cell volume^e^	13.5% (10.3, 17.6)	99.4% (99.3, 99.5)	32.4% (25.2, 40.5)	98.3% (98.0, 98.4)	4	67
	Any abnormal FBC	23.3% (16.7, 31.7)	99.3% (99.2, 99.4)	20.1% (14.3, 27.6)	99.4% (99.3, 99.5)	10	39
	Low serum ferritin^f^	9.6% (7.7, 11.8)	99.6% (99.5, 99.7)	55.4% (47.1, 63.4)	95.6% (95.3, 95.9)	6	62
	High serum ferritin^g^	17.0% (12.5, 22.6)	99.4% (99.2, 99.5)	40.0% (30.5, 50.3)	98.0% (97.7, 98.3)	15	98
	High C-reactive protein^h^	6.5% (2.2, 17.5)	99.0% (98.8, 99.2)	3.3% (1.1, 9.3)	99.5% (99.3, 99.6)	13	62

*Note*: Serum ferritin and c-reactive protein tests were only conducted for a subset of patients (*n* = 8,923 and 12,202 respectively)

*CI* Confidence interval

a <130 g/L in men and <120 g/L in women

b >400 μL/L

c >11,000/mL

d <27.4 pg/cell

e <80 fL

f <20 ng/mL

g ≥350 ng/mL

h >10 mg/L

At a threshold of 10 μg Hb/g faeces, 9.2% of patients would be considered FIT positive. Sensitivity was 92.1% (95% CI 86.4–95.5), specificity 91.5% (95% CI 91.1–91.9), PPV 8.4% (95%CI 7.1–9.9), and NPV 99.9% (95% CI 99.9–100) (Table 1, Additional file 1: Table S2). One cancer was detected for every twelve positive FITs, and a miss rate of 7 cancers per 10,000 negative tests (Table 2).

#### FIT-blood test pairs

Sensitivity ranged from 3.3% (FIT≥2 or 10 μg Hb/g faeces and raised CRP) to 56.8% (FIT≥2 μg Hb/g faeces and low serum ferritin) for pairings of FIT and blood tests. Specificity was higher for almost all pairings compared to a FIT-alone approach leading to fewer positives being needed to detect one cancer. However, the cancer miss rate per 10,000 tests increased 14-fold compared to a FIT alone approach (Table 2).

#### Multivariable FIT

A) Model A (with continuous FIT): sex and continuous variables for age, serum ferritin, platelets, and CRP were retained. Specificity was 45.9% (95% CI 44.7–47.1), compared to 90.0% for FIT alone (in the subset with serum ferritin and CRP), leading to one cancer in every 57 positive tests compared to one in 12 in the FIT-only approach (Table 3, Additional file 1: Table S2).
B) Model B (dichotomous FIT, blood tests): FIT, sex, and low MCV were retained. Specificity was 90.1% (95% CI 89.6–90.5), similar to FIT alone at FIT≥10 μg Hb/g faeces, leading to 14 positive tests to detect one cancer.
C) Model C (FIT spline): FIT, sex, and low MCV were retained. Specificity was 91.5% (95% CI 91.1–91.9) with one cancer detected for every 12 positive FITs.

**Table 3:** Test performance as measured by positive and negative predictive value (PPV, NPV), sensitivity, specificity, positive FITs per cancer detected, and cancer miss rate per 10,000 negative tests. FIT alone and model-based approach

Test criteria		PPV (95% CI)	NPV (95% CI)	Sensitivity (95% CI)	Specificity (95% CI)	Positive FITs to detect one cancer	Cancer miss rate per 10,000 negative tests
**FIT alone**							
FIT≥10 µg Hb/g		8.4% (7.1, 9.9)	99.9% (99.9, 100)	92.1% (86.4, 95.5)	91.5% (91.1, 91.9)	12	7
FIT≥10 µg Hb/g	In subset with serum ferritin & C-reactive protein^a^	8.8% (6.9, 11.1)	99.9% (99.8, 100)	93.8% (85, 97.5)	90.0% (89.2, 90.7)	11	7
**Multivariable model including FIT**							
**Model A**^**a**^**: **continuous FIT PLUS continuous variables selected by stepwise procedure: age, sex, serum ferritin, platelets, c-reactive protein		1.7% (1.4, 2.2)	99.9% (99.6, 99.9)	93.8% (85, 97.5)	45.9% (44.7, 47.1)	57	14
**Model B:** FIT≥10µg Hb/g PLUS categorical variables selected by stepwise procedure: sex, low mean cell volume		7.4% (6.2, 8.7)	99.9% (99.9, 100)	93.5% (88.2, 96.6)	90.1% (89.6, 90.5)	14	6
**Model C: **FIT Spline PLUS categorical variables selected by stepwise procedure: sex, low mean cell volume		8.4% (7.1, 9.9)	99.9% (99.9, 100)	92.1% (86.4, 95.5)	91.5% (91.1, 91.9)	12	7

In the model-based approach, a fixed threshold for a positive test was set to achieve the sensitivity of a FIT threshold of 10 in the FIT alone approach.

*CI* Confidence interval

^a^ Serum ferritin and c-reactive protein tests were only conducted for a subset of patients. The model was applied to patients with non-missing values for both tests

In summary, Models B and C performed similarly to FIT alone but no approach that integrated blood test results improved the overall performance of FIT. While FIT was always retained in stepwise selection irrespective of form, including the blood test variables in different forms (e.g., continuous vs. dichotomized) resulted in different variables being retained in the models. Odds ratios for the predictors and the log likelihood and area under the curve for each model are provided in Additional file 1: Table S3. A plot of apparent calibration did not reveal any causes for concern.

**Fig. 3:**
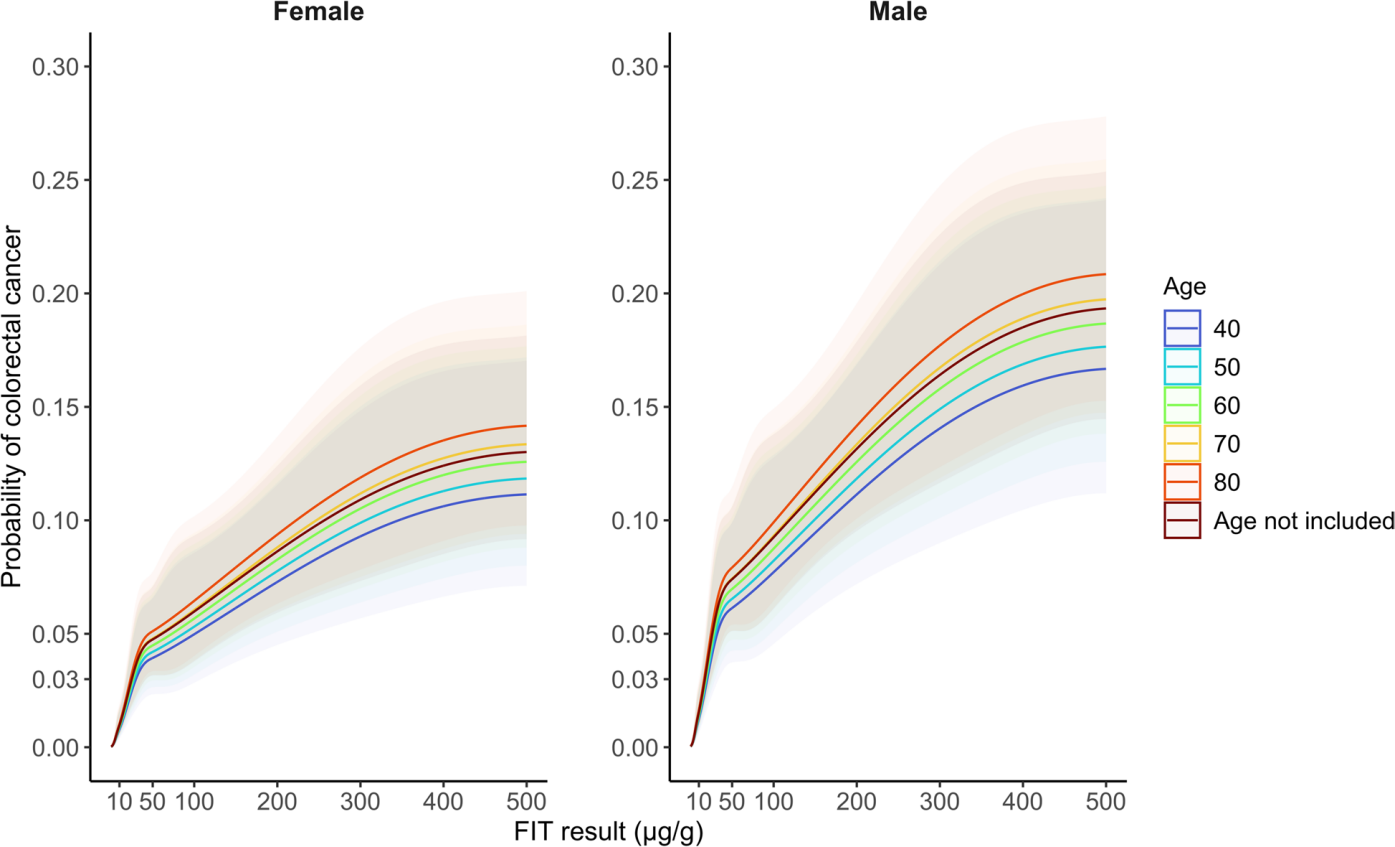
Probability of colorectal cancer by sex, age, and FIT score with 95% confidence intervals indicated with shading (See Model C). The restricted cubic spline function was specified to have knots at FIT values of 2, 10, 50, and 100

The age-specific probabilities of colorectal cancer by sex and FIT score based on Model C are illustrated in Fig. 3. For males and females, the probability of colorectal cancer reached 3% (the cut-off specified to prompt urgent investigation by NICE [22]) at FIT values of 17 and 25, respectively. There were no significant differences by age since age was not a significant predictor of cancer risk after accounting for FIT (Additional file 1: Table S3).

#### FIT-negative cancers

The characteristics of the 11 patients with false negative tests at a FIT threshold of 10 μg Hb/g faeces are provided in Table 3. Ten had at least one GP-reported clinical indication with the most common being change in bowel habit (*n* = 6). Eight of the 11 had at least one abnormal blood test with the most common being raised CRP (5 of 10 with known values). Median days from FIT to cancer diagnosis was 27 days among false negatives (interquartile range 21 to 55) compared to 34 (21, 64) among persons diagnosed with cancer overall (Table 4).

**Table 4:**
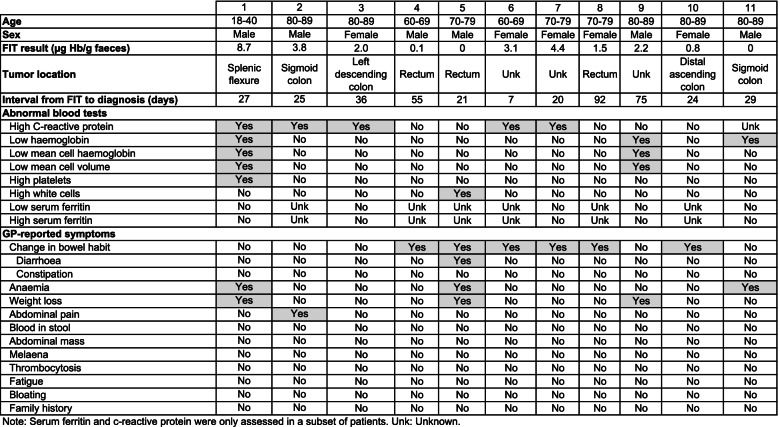
Clinical characteristics of patients who had a false-negative FIT at a threshold of 10 μg Hb/g faeces

#### Subgroup and sensitivity analyses

Patient demographics, clinical indication, prevalence of abnormal blood tests, FIT score, and performance of FIT were largely consistent prior to and during the COVID-19 pandemic (Additional file 1: Tables S4 and S5). The median age of persons undergoing FIT was older during COVID (64 vs. 59 years), but the interquartile range was similar (51 to 76 vs. 51 to 74). There were no significant differences in sensitivity, specificity, PPV, or NPV of FIT.

PPV was higher among males than females, but the confidence intervals for the two sexes overlapped at a threshold of 10 μg Hb/g faeces. At 2 μg Hb/g faeces, PPV and NPV decreased with increasing age. At 10 μg Hb/g faeces, PPV and NPV were largely consistent by age group (Additional file 1: Table S6).

There was no evidence that the PPV of FIT was significantly higher within subgroups defined by symptoms at presentation or blood test other than MCV (Additional file 1: Table S6). Sensitivity increased and specificity decreased in subgroups defined by increasingly severe anaemia (Additional file 1: Table S7). Fifty-seven percent of patients met the criteria for FIT under the DG30, which specifies use for patients without rectal bleeding and specific symptoms depending on age [2]. The incidence of cancer was slightly lower in the DG30-qualifying group (0.8 vs. 0.9%), and specificity was marginally higher (84.3 vs. 82.5% in other, Supplementary Table 8). Results did not meaningfully change when the follow-up period was extended to 12 months (Additional file 1: Tables S9 and S10). The positive predictive value of an abnormal blood test in the FIT-negative population was consistently less than 1% (Additional file 1: Table S11).

## Discussion

### Statement of principal findings

In this large cohort of patients tested with FIT in primary care, neither age, nor blood test results remained strong enough predictors of colorectal cancer to improve on the performance of FIT. While the number of false positives could be reduced by taking into account blood tests, the large associated increase in false negatives outweighed the benefit. In addition, there was no evidence to suggest that using clinical indication as a rule-out or rule-in factor would improve the efficiency of FIT triage. The lack of an apparent age-effect after taking into account FIT suggests that age-specific thresholds for FIT positivity would not improve test performance.

### Strengths and weaknesses of the study

This is the largest cohort of primary care patients tested with FIT in the UK. The sample comprises patients reflecting true clinical practice which involves uptake of the DG30 guideline over time and clinical judgement. The study also comprises tests prior to and during the COVID-19 pandemic and suggests that the performance of FIT in primary care has remained stable throughout. Centralised FIT and blood testing at the the OUH Clinical Biochemistry Laboratory allowed for highly complete assessment of FIT and blood test values. We accessed the referral text to explore the performance of FIT in strata defined by GP-reported symptoms. The prevalence of and type of symptoms reported may have differed if we had accessed primary care records or asked patients to report their symptoms directly [23]. A 6-month follow-up period was used for the primary analysis to optimise the number of cancers included, but in sensitivity analyses that aligned with national guideline-setting, a 12-month follow-up showed similar results.

With respect to limitations, the gold standard would have been to have every patient undergo a colonoscopy after FIT. Due to the observational nature of this study, we instead used hospital-based records to determine outcomes after FIT. This may have resulted in some underestimation of disease. However, by linking multiple local data sources for patients tested in a single central laboratory in a clearly defined geographical catchment area we increased the likelihood that serious disease diagnosed during the study period was captured. It is plausible that some patients may have been lost to follow-up by moving out of area or by dying, but this is unlikely to have had a meaningful impact on our findings, as we used a short follow-up period and there is minimal patient movement between localities, particularly during investigation. An alternative approach would have been linkage to Public Health England’s National Cancer Registration and Analysis Service (NCRAS). While this may have provided a more complete accounting of outcomes, it would have restricted the timeliness of our analyses as cancer registry data is currently available only up until the end of 2018. Taking into account the follow-up period, this would have limited us to fewer than 4000 FITs for inclusion.

Another potential limitation is that the sample was restricted to individuals for whom blood test results were available within a 90-day window surrounding FIT, but this excluded only 10% of the sample. As no predictive model was identified that performed better than FIT alone, neither internal validation nor optimism correction were pursued.

### Comparison with existing literature

Recent studies have reported FIT to be an effective tool to triage for “high-risk” patients referred for definitive investigation [9, 13]. Our findings contribute to a growing literature suggesting that FIT also performs well in the “lower risk” primary care setting [6, 7, 16, 24, 25]. This is the one of few studies to formally and systematically evaluate blood tests in addition to FIT in symptomatic patients, and one of few to analyse FIT supplemented with other variables. The f-Hb, age, and sex test score (FAST) was not superior to FIT alone in the primary care setting [12]. COLONPREDICT included FIT, age, sex, rectal bleeding, benign anorectal lesions, rectal mass, serum carcinoembryonic antigen, blood haemoglobin, colonoscopy in the last 10 years, and treatment with aspirin. COLONPREDICT was derived and validated in a higher-risk referred population [26] and at a threshold equivalent to approximately 90% sensitivity (f-Hb≥20 μg Hb/g faeces and COLONPREDICT score≥5.6) had a specificity of 78.7% compared to 69.6% in FIT alone [27].

A UK-based study of whether demographic, lifestyle (e.g., smoking, physical activity), or clinical factors (family history, symptoms) could add to the predictive value of FIT found that only family history of polyps showed a significant association once FIT was taken into account [28]. In the current study, family history was not retained in stepwise models; however, the indicator was based on referral notes whereas in the aforementioned study, patients were prospectively asked about family history.

### Unanswered questions and future research

FIT is a sensitive and specific test and as such can serve as a valuable rule-in and rule-out test for patients presenting to primary care. However, it remains worthwhile to investigate strategies to further enhance the sensitivity and specificity of FIT and guide prioritisation of FIT-positive patients for immediate colonoscopy. Risk stratification tools that have been developed in the screening setting incorporating polygenic risk scores [29–31], urinary volatile organic compounds [10, 32], and circulating and/or faecal tumour DNA [33] could be explored to complement FIT for triage of primary care patients.

Currently, there is limited evidence to support the use of repeat FIT testing to select initially FIT-negative patients for referral or to reassure about non-referral. Based on exploratory analyses, 1113 patients in our study had second FITs also meeting inclusion criteria. In that group, there were 6 cancers, and no false negatives at a threshold of 2 or 10. Of the 1007 patients without cancer, forty-four (4.3%) patients had initially negative FITs followed by false-positive FITs at a threshold of 10, and 942 (93.5%) participants without cancer had two negative FITs. Further research is needed to inform the timing, interpretation, and utility of repeat FIT testing for triage in symptomatic patients attending primary care.

Both FIT and the “gold standard” colonoscopy result in false negatives [34]. In this study, no practical rules using blood tests or clinical indication to reduce false negatives were apparent. To reduce the likelihood of false FIT-negative results, future research may benefit from an agnostic approach to building the prediction model. For example, new predictive markers could be discovered by applying machine learning models to large, representative databases of electronic health records [35].

## Conclusions

FIT alone is simple, easily implemented and effective to triage patients from primary care to colonoscopy. Particularly in light of the COVID pandemic and the suspected accumulation of undiagnosed cancers and unscreened adults [11], effective methods to triage low and/or “intermediate” risk patients to referral are more needed than ever [36]. Our results suggest however, that neither age, nor blood tests, nor clinical indication as recorded by the physician should be used to inform referral to colonoscopy after FIT. We found that the performance of FIT was maintained in patients with increasingly severe anaemia supporting that FIT can still be used in this group prior to referral for secondary care.

In the absence of alternate strategies to complement FIT, follow-up care of FIT-negative patients should focus on safety netting, including the re-evaluation of patients with persistent and unexplained symptoms within a pre-specified timeframe in primary care, and the possibility of urgent specialist assessment of FIT-negative patients for whom there is ongoing clinical concern [37–39].

## Supplementary Information

**Additional file 1: Table S1.** [Frequency of abnormal blood test results by outcome and FIT score]. **Table S2.** [Distribution of test results within study population by approach]. **Table S3.** [Model parameters for models resulting from backward stepwise regression]. **Table S4.** [Characteristics of patients receiving symptomatic FIT tests by date of FIT relative to COVID-19 pandemic]. **Table S5.** [Test performance comparing pre and post COVID19]. **Table S6.** [Test performance by demographic and clinical subgroups]. **Table S7.** [Test performance by subgroups defined by increasingly severe anaemia]. **Table S8.** [Test performance comparing patients who meet the DG30 criteria]. **Table S9.** [Test performance with 12 months of follow-up, FIT alone and FIT-blood test pairs]. **Table S10.** [Test performance with 12 months of follow-up, FIT alone and model-based approach.]. **Table S11.** [Predictive performance of abnormal blood tests among patients who are FIT negative].

## Data Availability

The datasets analysed during the current study were not collected for research purposes and are not publicly available due to anonymity reasons.

## Abbreviations

BRC: Biomedical Research Center
CRP: C-reactive protein
CV: Coefficient of variation
FAST: Faecal haemoglobin Age and Sex Test
FIT: Faecal immunochemical test
GP: General practitioner
MCH: Mean cell haemoglobin
MCV: Mean cell volume
NICE: National Institute for health and Care Excellence
NIHR: National Institute for Health Research
NPV: Negative predictive value
OUH: Oxford University Hospitals
PPV: Positive predictive value
