# Supplementary material for: Combining faecal immunochemical testing with blood test results for colorectal cancer risk stratification: a consecutive cohort of 16,604 patients presenting to primary care
Source: BMC Med. 2022 Mar 15;20:116. doi: 10.1186/s12916-022-02272-w (PMC8920746; doi:10.1186/s12916-022-02272-w)
Supplement: Supplementary file 1 — Additional file 1: Table S1. [Frequency of abnormal blood test results by outcome and FIT score]. Table S2. [Distribution of test results within study population by approach]. Table S3. [Model parameters for models resulting from backward stepwise regression]. Table S4. [Characteristics of patients receiving symptomatic FIT tests by date of FIT relative to COVID-19 pandemic]. Table S5. [Test performance comparing pre and post COVID19]. Table S6. [Test performance by demographic and clinical subgroups]. Table S7. [Test performance by subgroups defined by increasingly severe anaemia]. Table S8. [Test performance comparing patients who meet the DG30 criteria]. Table S9. [Test performance with 12 months of follow-up, FIT alone and FIT-blood test pairs]. Table S10. [Test performance with 12 months of follow-up, FIT alone and model-based approach.]. Table S11. [Predictive performance of abnormal blood tests among patients who are FIT negative]. [file 12916_2022_2272_MOESM1_ESM.docx]

**ADDITIONAL FILE 1**
